# Expanding the Role of Mindfulness in IBD Care: Bridging Psychological Well-being and Disease Management

**DOI:** 10.62641/aep.v53i1.1836

**Published:** 2025-01-05

**Authors:** Guilherme Nobre Nogueira

**Affiliations:** ^1^Internal Medicine Department, UFC - Universidade Federal Do Ceará, 60455-760 Fortaleza, Ceará, Brazil

Dear Editor,

I am writing to express my sincere appreciation for the insightful article titled “*Mindfulness-Based Interventions on Psychological Comorbidities in Patients with Inflammatory Bowel Disease: A Systematic Review and Meta-Analysis*” by Qian and Zhang [1]. This comprehensive study offers a critical perspective on the role of mindfulness practices in enhancing mental health and overall quality of life for individuals suffering from inflammatory bowel disease (IBD).

The findings of this review are especially relevant in light of the growing acknowledgment of the complex relationship between psychological well-being and chronic physical conditions [2, 3]. The authors’ systematic and rigorous approach to analyzing the literature, including thorough screening and data extraction methods, lends substantial credibility to their conclusions. Their meta-analysis clearly demonstrates the positive impact of mindfulness-based interventions in alleviating anxiety and depression among IBD patients—an essential component of holistic patient care that often remains underemphasized.

However, I would like to suggest a few considerations that could deepen the discussion on this crucial topic. While the study highlights the overall benefits of mindfulness, it would be advantageous to explore in greater detail the underlying mechanisms through which these interventions exert their psychological and physiological effects. Unpacking the neurobiological and psychobehavioral pathways—such as the modulation of the hypothalamic-pituitary-adrenal (HPA) axis or reductions in inflammation—could further solidify the case for incorporating mindfulness into IBD treatment protocols. This exploration could also inform future research on how mindfulness may mitigate disease progression or flare-ups linked to psychological stress [4].

Additionally, the review points out the heterogeneity in the mindfulness interventions employed across the studies, such as variations in duration, frequency, and specific techniques used. This variability poses challenges when recommending particular practices. Future research would greatly benefit from standardized intervention protocols , which would allow more accurate efficacy comparisons across different mindfulness techniques. Establishing which practices are most effective for IBD patients—and under what circumstances—would offer valuable guidance for clinical application.

Moreover, I believe the implications for clinical practice warrant further discussion. With the increasing trend towards integrative healthcare approaches, mindfulness training holds promise as a complementary therapy within IBD care [5]. Incorporating structured mindfulness programs into standard treatment regimens could provide a non-pharmacological option to enhance mental resilience, reduce symptom burden, and improve overall quality of life. Such integration could also empower patients to take an active role in managing their condition’s psychological and physical aspects.

In conclusion, I commend Qian and Zhang [1] for their significant contribution to the understanding of the interplay between psychological comorbidities and chronic illness, particularly IBD. Their work paves the way for further research and clinical application of mindfulness-based interventions in managing chronic disease’s mental and physical dimensions.

## Availability of Data and Materials

Not applicable.
